# Response to the Letter: “Enhancing the Understanding of Paternal Involvement in Childcare and Its Impact on Maternal Disciplinary Practices”

**DOI:** 10.2188/jea.JE20240433

**Published:** 2025-05-05

**Authors:** Mako Nagayoshi, Yuko Kachi, Tsuguhiko Kato, Manami Ochi, Yuichi Ichinose, Takayuki Kondo, Kenji Takehara

**Affiliations:** 1 Department of Preventive Medicine, Nagoya University Graduate School of Medicine, Nagoya, Japan; 2 Department of Social Medicine, National Center for Child Health and Development, Tokyo, Japan; 3 Division of Health Policy and Management, Graduate School of Public Health, St. Luke’s International University, Tokyo, Japan; 4 Department of Health Policy, National Center for Child Health and Development, Tokyo, Japan; 5 Department of Health and Welfare Services, National Institute of Public Health, Saitama, Japan; 6 Division of Health Services Research, Institute for Cancer Control, National Cancer Center, Tokyo, Japan; 7 Center for Research on Poverty among Children and Youth, Tokyo Metropolitan University, Tokyo, Japan

**Keywords:** paternal behavior, childrearing, housework, punishment

We appreciate the detailed comments on our manuscript provided by Hong Pan. This letter highlights the importance of fatherhood in family ethics and the impact of social environment on them, as shown in the paper by Nagayoshi et al.^1^ The letter proposes three points for future research: 1) conducting surveys involving a broader populace to grasp the complexity of situations in families and societies; 2) increasing survey participation rates by reducing the burden on respondents; and 3) exploring factors beyond fathers’ involvement in childcare and housework that could reduce childcare stress.

First, we agree that verification among a diverse population is essential to understand the complexity of the situation in families and societies. The Japanese Longitudinal Survey of Newborns in the 21st century (JLSN21), the data used in our study,^1^ is conducted under the Statistics Law of Japan.^2^ The target population of the survey was all infants born in Japan between May 10 and 24, 2010 (*n* = 43,767) and their families,^2^^,^^3^ offering comprehensive coverage. However, even with a broader population, variables based on diversity may not suffice. In our study,^1^ we only focused on the frequency of fathers’ involvement in childcare and housework and fathers’ working hours. As pointed out by Hong Pan, the dynamics within the family, cultural background, and social supports were not verified. Therefore, verifying the influence of these factors on the outcomes among a diverse group of people is necessary.

Second, efforts should be made to increase survey participation. Although the response rate of the JLSN21 was relatively high, at around 90%,^3^ maintaining a high follow-up rate is difficult because participation declines overtime. While, the fact that limiting the analytic sample to mothers had a greater impact on the reduced sample size.^1^ Although the JLSN21 allowed any household adult—mothers, fathers, or grandparents—to respond, we restricted it to mothers to standardize perspectives on fathers’ involvement.^1^^,^^4^ Additional restriction to mothers who responded to both surveys (the first and fourth surveys) reduced the sample size to 42% of the original target population.^1^ Reducing the burden on respondents is important to improving participation. Apart from simplifying questionnaires or conducting phased surveys, adopting new technologies may improve participation. In the JLSN21, the response rate improved after the 10th survey, which was available online.^3^

Third, evaluating additional factors, such as supports beyond families, will be essential, as noted in the letter. As the burden of childrearing largely differs by availability of supports from community, friends, and other family members, such information enables us to identify mothers who need supports. This perspective is particularly important in Japan, where the demographic situation is changing dramatically. With the hyperaged society, where 29.1% of the population are elderly (65 years or older) and only 18.1% of households have children younger than 18 years, families with children are rare. Additionally, the rise of nuclear families (about 88% in 2020) and dual-income households (about 70% in 2022) further complicates understanding of the challenges faced by families with children. Such situations may be making it harder for mothers to seek mutual support within their communities.

Future studies should verify and expand the findings of our study, focusing on broader cultural and social contexts. Government statistical data, such as the JLSN21, will be increasingly important for future analyses. Currently, two birth cohorts have continued since 2001 and 2010. Even within this short timeframe, the environment for children and parents in Japan has changed considerably, including a decrease in mothers’ frequent spanking behavior (from 9.9% in the first cohort to 5.2% in the second).^4^ Since 2010, there have been further cultural and social shifts, such as changing attitudes toward gender-specific roles among younger generations and policy developments, like the April 2022 enactment of laws to promote paternity leave. These changes underscore the importance of creating a new birth cohort to understand their impact on family dynamics and society. We hope that continued research, including updated analysis of new birth cohorts, will lead to a better environment for parents to raise their children. Such structural changes enable men to more easily take on childcare and housework responsibilities.

Once again, we express our gratitude toward the Letter by Hong Pan; their constructive comments for our paper were truly encouraging.

## References

[1] Nagayoshi M, Kachi Y, Kato T, . Paternal involvement in childcare and housework and mothers’ spanking behavior: the Japanese longitudinal survey of newborns in the 21st century. J Epidemiol. 2024;34(12):577–586. 10.2188/jea.JE20230270 38853010 PMC11564066

[2] Ministry of Health, Labour and Welfare. The Japanese Longitudinal Survey of Newborns in the 21st century (Newborns in 2010). https://www.mhlw.go.jp/toukei/list/27-22.html (accessed on November 13, 2024).

[3] Ministry of Health, Labour and Welfare. Overview of the Japanese Longitudinal Survey of Newborns in the 21st century (Newborns in 2010) 2023. https://www.mhlw.go.jp/toukei/saikin/hw/syusseiji/22/index.html (accessed on November 13, 2024).

[4] Baba S, Eshak ES, Shirai K, Fujiwara T, Yamaoka Y, Iso H. Factors associated with family member’s spanking of 3.5-year-old children in Japan. J Epidemiol. 2020;30(10):464–473. 10.2188/jea.JE20190160 31685725 PMC7492701

